# Familial, structural, and environmental correlates of MRI-defined bone marrow lesions: a sibpair study

**DOI:** 10.1186/ar2027

**Published:** 2006-08-03

**Authors:** Guangju Zhai, James Stankovich, Flavia Cicuttini, Changhai Ding, Graeme Jones

**Affiliations:** 1 Menzies Research Institute, University of Tasmania, Level 2, Surrey House, 199 Macquarie Street, Hobart, TAS 7000, Australia; 2 Twin Research and Genetic Epidemiology Unit, St Thomas's Hospital, Lambeth Palace Road, London, SE1 7EH, UK; 3 The Walter and Eliza Hall Institute of Medical Research, 1G Royal Parade, Parkville, Melbourne, VIC 3050, Australia; 4 Department of Epidemiology and Preventive Medicine, Monash University Medical School, 89 Commercial Road, Alfred Hospital, Melbourne, VIC 3004, Australia

## Abstract

The aim of this study was to estimate the heritability and describe the correlates of bone marrow lesions in knee subchondral bone. A sibpair design was used. T2- and T1-weighted MRI scans were performed on the right knee to assess bone marrow lesions at lateral tibia and femora and medial tibia and femora, as well as chondral defects. A radiograph was taken on the same knee and scored for individual features of osteoarthritis (radiographic osteoarthritis; ROA) and alignment. Other variables measured included height, weight, knee pain, and lower-limb muscle strength. Heritability was estimated with the program SOLAR (Sequential Oligogenetic Linkage Analysis Routines). A total of 115 siblings (60 females and 55 males) from 48 families, representing 95 sib pairs, took part. The adjusted heritability estimates were 53 ± 28% (mean ± SEM; *p *= 0.03) and 65 ± 32% (*p *= 0.03) for severity of bone marrow lesions at lateral and medial compartments, respectively. The estimates were reduced by 8 to 9% after adjustment for chondral defects and ROA (but not alignment). The adjusted heritability estimate was 99% for prevalent bone marrow lesions at both lateral and medial compartments. Both lateral and medial bone marrow lesions were significantly correlated with age, chondral defects, and ROA of the knee (all *p *< 0.05). Medial bone marrow lesions were also more common in males and were correlated with body mass index (BMI). Thus, bone marrow lesions have a significant genetic component. They commonly coexist with chondral defects and ROA but only share common genetic mechanisms to a limited degree. They are also more common with increasing age, male sex, and increasing BMI.

## Introduction

Osteoarthritis (OA) is the most common form of arthritis, especially of the knee, and is a leading cause of musculoskeletal disability in most developed countries [1]. Although the exact pathogenesis remains unknown, OA of the knee is believed to be multifactorial and involves the whole joint. Felson and colleagues [2] first demonstrated that bone marrow lesions observed by MRI were associated with the presence of pain in OA of the knee, indicating its clinical significance. However, there are limited data on their pathology and causes. Altered biomechanical stress can cause similar bone marrow lesions in the feet, knee and hip of healthy subjects [3], whereas running can cause similar lesions in the foot and ankle [4], implying that altered loading across bones might be a possible cause of bone marrow lesions. Indeed, knee alignment is one of the key determinants of load distribution [5], and knee medial bone marrow lesions are more likely in OA patients with varus knee alignment, whereas lateral bone marrow lesions are more common in those with valgus alignment [6]. Chondral defects and bone marrow lesions commonly coexist in patients with either OA or chondral injuries, and bone marrow lesions are mostly located beneath chondral defects [7-9]. However, we recently found in a large sample that chondral defects and bone marrow lesions were independently associated with knee pain [10], suggesting other pathways between bone marrow lesions and pain.

In a previous study, we reported that knee cartilage volume, bone size and chondral defects all have high heritability, suggesting their potential for association and linkage studies [11,12]. With the use of the same sibpair cohort measured at follow-up, the aim of the present study was to estimate the heritability of bone marrow lesions and to assess whether the heritability is independent of other factors including chondral defects and knee alignment. Further, we describe the correlates of bone marrow lesions with both structural and environmental factors measured in the study.

## Materials and methods

### Study subjects

The study was performed in Southern Tasmania as described previously [13]. In brief, subjects were the adult children of patients who had had a knee replacement performed for idiopathic OA of the knee. The subjects were followed up for two years. At the follow-up, all participants were assessed for bone marrow lesions. The Southern Tasmanian Health and Medical Human Research Ethics Committee approved the study and written informed consent was obtained from all participants.

### Anthropometrics

Weight, height and muscle strength were measured as described previously [13]. Knee pain was assessed by self-administered questionnaire using the Western Ontario and McMaster Universities Osteoarthritis Index (WOMAC) [14]. Five categories of pain (walking on flat surface, going up or down stirs, at night, sitting or lying, and standing upright) were assessed separately with a 10-point scale from 0 (no pain) to 9 (most severe pain). Each score was then summed to create a total pain score (range 0 to 45).

### Magnetic resonance imaging

An MRI scan of the right knee was performed at the follow-up. Knees were imaged in the sagittal plane on a 1.5-tesla whole-body magnetic resonance unit (Picker, Cleveland, OH, USA) with the use of a commercial transmit–receive extremity coil. The following image sequence was used: a T2-weighted fat saturation two-dimensional fast spin echo; flip angle 90°; repetition time 3,067 ms echo time 112 ms; field of view 16 cm/15 partitions; 228 × 256 matrix; sagittal images were obtained at a partition thickness of 4 mm with a between-slices gap of 0.5 to 1.0 mm.

Subchondral bone marrow lesions were assessed on these serial MR images and defined as discrete areas of increased signal adjacent to the subcortical bone at lateral tibia and/or femora, medial tibia and/or femora. Each bone marrow lesion was scored on the basis of lesion size as described previously [10]. A lesion was scored as grade 1 if it was present only on one slice, grade 2 if on two consecutive slices, or grade 3 if on three or more consecutive slices. The highest score was used if more than one lesion was present on the same site. Summation of the score was regarded as an indication of severity of bone lesions, while prevalent bone marrow lesions were defined as a total score of 1 or more. One observer (GZ) scored the bone marrow lesions, blinded to other variables. Intra-observer repeatability was assessed in 50 subjects with at least a 1-week interval between two readings with intraclass correlation coefficients (ICCs) of 0.89 to 1.00.

In addition, T1-weighted fat saturation three-dimensional SPGR (Spoiled Gradient Recalled Acquisition in the Steady State) MRI scans were also performed on the same knee at the follow-up. Chondral defects were assessed on these images and scored with a modification of a previous classification system [15] at medial tibial, medial femoral, lateral tibial and lateral femoral sites as follows: grade 0 = normal cartilage; grade 1 = focal blistering and intracartilaginous low-signal intensity area with an intact surface; grade 2 = irregularities on the surface or basal layer and loss of thickness less than 50%; grade 3 = deep ulceration with loss of thickness more than 50%; and grade 4 = full-thickness chondral wear with exposure of subchondral bone. We found that cartilage surface in some images was still regular but cartilage adjacent to subchondral bone became irregular, so we included these changes in the classification system. A cartilage defect also had to be present in at least two consecutive slices. The cartilage was considered to be normal if the band of intermediate signal intensity had a uniform thickness. The highest score was used if more than one defect was present on the same site. Two observers (CD and HC) scored the MRI blind to bone marrow lesions and other clinical information. Interobserver reliability was assessed in 50 individual magnetic resonance images and yielded an ICC of 0.89 to 0.93 for different compartments. Intraobserver reliability in the whole sample (expressed as ICC) was 0.92 to 0.94. Chondral defects were defined as presence of the disease (a score of 2 or more) and the total score (0 to 8) for lateral and medial compartments, respectively.

### X-rays

A standing AP semiflexed view of the right knee was performed in all subjects at baseline and assessed according to the Altman atlas [16]. Each of the following was assessed on a scale of 0 to 3 for increasing severity: medial joint space narrowing (JSN), lateral JSN, medial osteophytes (femoral and tibial combined), and lateral osteophytes (femoral and tibial combined). Each score was arrived at by consensus, with two readers (GJ and FS) simultaneously assessing the radiograph with immediate reference to the atlas. Radiographic osteoarthritis (ROA) was defined by the presence of disease (a score of more than 0) and total score (0 to 12). Reproducibility was assessed in 50 radiographs 2 weeks apart, yielding an ICC of 0.99 for osteophytes and 0.98 for JSN.

Knee alignment was also measured on the same knee radiograph by using a method validated previously [17,18]. Lines were drawn through the middle of the femoral shaft and through the middle of the tibial shaft. The angle subtended at the point at which these two lines met in the centre of the tibial spines was measured by a protractor (Protractor Stirflex Pro; ORNA IPLAST S.p.A., Cavaion, Verona, Italy) manually on the X-ray. The measurement was done by a single observer (GZ). The intra-observer reproducibility was assessed in 30 subjects with two measurements at least one month apart. The ICC was 0.97.

### Statistics

A variance components analysis was performed to estimate the heritabilities of various traits. With the use of the software package SOLAR (Sequential Oligogenetic Linkage Analysis Routines) [19], trait variance was modelled as a mixture of genetic variance (attributed to many genes with small, additive effects) and random variance (due to random environmental variations not correlated between subjects within families). Then the estimated heritability was defined as the proportion of genetic variance in the model with the maximum likelihood.

To assess whether the estimated heritabilities differed significantly from zero, a null model with only the random variance term was also fitted. All models were fitted after first adjusting trait scores within SOLAR for various combinations of covariates: first, age, sex, height and weight; second, all previous covariates, knee pain, muscle strength and knee alignment; third, all previous covariates and chondral defects score; and fourth, all previous covariates and ROA. Spearman's correlation coefficient was used for examining the correlation between bone marrow lesions and factors of interest. A *p *value of less than 0.05 was regarded as statistically significant.

## Results

A total of 115 subjects (55 males and 60 females) representing 95 sib pairs with an average age of 47 years took part in this study. Thirty-five families had two children, nine had three, three had four, and one had six. Table 1 presents the characteristics of the subjects. The prevalence of bone marrow lesions was 14% and 24% for lateral and medial compartments, respectively, but most were mild as indicated by a mean total score of 0.27 to 0.48 (SD 0.78 to 1.09). The prevalence of grade 1 bone marrow lesions was 6% and 10% for lateral and medial compartments, respectively, and accounted for 40% of the total prevalence. Medial bone marrow lesions were more common in males (*p *= 0.04). Chondral defects and knee pain were also mild, and ROA was relatively uncommon at baseline. Knee alignment was 180.4°, with a low SD of ± 2.6°.

Both lateral and medial bone marrow lesions were significantly correlated with age (Spearman's rho = 0.26 and 0.27, respectively; *p *< 0.01 for both), chondral defects (Spearman's rho = 0.26 for both; *p *< 0.01) and knee ROA (Spearman's rho = 0.20 and 0.23; *p *= 0.04 and 0.02, respectively). Medial bone marrow lesions were also correlated with body mass index (BMI; Spearman's rho = 0.19; *p *= 0.04). No association was observed for previous knee injury, knee alignment and muscle strength.

Table 2 presents the heritability estimates for bone marrow lesions. The heritability estimates were significant for both severity and prevalence of bone marrow lesions at both lateral and medial compartments after adjustment for age, sex, height, weight, muscle strength, knee pain and knee alignment. There was an 8 to 9% reduction in the estimate for the severity but only a 1% reduction for prevalence after adjustment for chondral defects and ROA, and the estimates remained significant or borderline significant. The heritability estimate for knee alignment was zero.

## Discussion

This is, to our knowledge, the first study that reports on causes of bone marrow lesions and documents a genetic contribution to both the prevalence and severity of bone marrow lesions in subchondral knee bone. The heritability estimates were reduced by a small amount after adjustment for chondral defects and ROA, suggesting that they share common genetic mechanisms to only a limited degree. The heritability estimate for knee alignment was zero, suggesting that it is not a heritable trait. Bone marrow lesions were also associated with some structural change within the knee and have some risk factors in common with osteoarthritis.

MRI-defined bone marrow lesions were first described by Wilson and colleagues [20] in patients with debilitating knee and hip pain. Felson and colleagues [2] documented its clinical relevance to pain in OA of the knee. Sower and colleagues [8] reported that women with bone marrow lesions and full-thickness chondral defects accompanied by adjacent subchondral cortical bone defects were significantly more likely than others to have painful OA of the knee. In a recent study of an older population [10], we demonstrated that ROA was not independently associated with knee pain but MRI-defined bone marrow lesions were associated with knee pain independently of ROA and chondral defects, suggesting an independent effect and wider clinical relevance. However, both the pathology and causes of MRI-defined bone marrow lesions are unknown. Felson and colleagues [6] reported that medial bone marrow lesions were more likely in OA patients with varus limbs, whereas lateral lesions were seen mostly in those with valgus limbs. Malalignment mediated 37 to 53% of the association between bone marrow lesions and progression of OA of the knee, suggesting that knee alignment may have a role in the occurrence of bone marrow lesions.

The current study is the first to document a significant genetic contribution, suggesting that further studies to identify specific gene(s) responsible for the development of bone marrow lesions might shed light on the prevention and management of knee pain. The heritability estimate was high for prevalent bone marrow lesions and independent of other factors including knee pain, knee alignment, chondral defects, and ROA, suggesting that they are under independent genetic control, with at most a small shared genetic component. However, the inability to estimate the standard error for the prevalence heritability estimates indicates that the results are not robust, possibly reflecting relative limitations of the program we used for dichotomous traits in comparison with continuous traits [21]. It is likely that the true heritability is substantially lower.

In comparison with prevalent bone marrow lesions, the heritability estimate for severity of bone marrow lesions was lower, but with a smaller standard error. The estimate again remained significant after adjustment for other factors including knee pain, muscle strength and knee alignment, suggesting that they are not under common genetic control. However, the estimate was reduced by 8 to 9% after adjustment for chondral defects and ROA, suggesting that they share common genetic mechanisms to a limited degree.

In contrast to this, but consistent with previous reports [7-9], was our observation that bone marrow lesions coexist with chondral defects and ROA of the knee, suggesting that they have environmental factors in common. Significant correlations between bone marrow lesions, age and BMI in the current study support this, although the increased prevalence in males suggests a possible role for trauma. However, in contrast to other reports [6,22], we did not find a significant association between knee alignment and bone marrow lesions, possibly because of a low prevalence of ROA in this sample. Further studies with independent samples are needed to confirm these results and confirm whether bone marrow lesions independently predict cartilage loss as chondral defects do [23].

The current study has several potential limitations. First, there is controversy about the ideal study design for estimating the heritability of disease. The twin model is often used but has been criticized as overestimating heritability because of the assumption of similar shared environments between monozygotic and dizygotic twins. This has been documented for bone mineral density [24] but not for osteoarthritis. Family studies such as the present one may be more likely to represent true heritability but make it more difficult to assess the contribution of shared environment. However, before this study, little was known about environmental effects on bone marrow lesions and we adjusted for all significant covariates in the analysis, so the results do not support a strong shared environmental contribution.

Second, the choice of subjects who are at all at higher risk of disease may bias the heritability estimates and limit the generalizability of the results to the general population. However, it is most likely that this bias will act to decrease estimates by decreasing genetic heterogeneity in comparison with an unselected sample.

Third, the bone marrow lesions were assessed in only one plane and the scoring system may not differentiate between various sizes of lesions in sagittal plane. However, most lesions are spherical, which suggests that they will have the same anteroposterior and lateral dimensions and would be strongly correlated with a volumetric scoring system based on mathematical principles [22]. Measurement error in the assessment of bone marrow lesions may have reduced the estimates. However, the method had high intra-observer reproducibility and we used a single observer for all readings, suggesting that this is not of major concern.

Fourth, using baseline X-ray measurements may not be appropriate because there was a two-year gap between the X-ray and MRI measurements. However, there is little radiographic change over this time frame and within-subject correlation for X-ray changes is very high, suggesting that this is not a big concern.

Fifth, bone marrow lesions in this sample were generally mild with grade 1 lesions accounting for 40% of the total prevalence, raising a concern of clinical relevance. However, these lesions have been associated with knee pain [2,10], suggesting that they are still clinically relevant.

Last, a clear elucidation of the nature of MRI-defined bone marrow lesions is uncertain. In a histological study of specimens taken from end-stage knees undergoing total joint replacement, Zanetti and colleagues [25] reported histological evidence of fibrosis, marrow necrosis and abnormal trabeculae for MRI-defined bone marrow lesions.

## Conclusion

This study demonstrates that bone marrow lesions have a significant genetic component. They commonly coexist with chondral defects and ROA but share common genetic mechanisms to only a limited degree. They are also more common with increasing age, male sex and increasing BMI.

## Abbreviations

BMI = body mass index; ICC = intraclass correlation coefficient; JSN = joint space narrowing; MRI = magnetic resonance imaging; OA = osteoarthritis; ROA = radiographic osteoarthritis; SOLAR = Sequential Oligogenetic Linkage Analysis Routines.

## Competing interests

The authors declare that they have no competing interests.

## Authors' contributions

GJ, GZ and FC were responsible for the study design and interpretation of the results. CD and GZ performed data collection. GZ, JS and GJ conducted the statistical analysis. GZ and GJ prepared the manuscript, with critical suggestions and comments from FC, CD and JS. All authors read and approved the final manuscript.

## Figures and Tables

**Table 1:** Characteristics of the subjects

Characteristic	Value (*n *= 115)
Age (years)	47 ± 6.9
Female sex (%)	52
Height (cm)	168.9 ± 8.9
Weight (kg)	80 ± 16.4
Lateral BML total score (possible range 0–6)	0.27 ± 0.78
Medial BML total score (possible range 0–6)	0.48 ± 1.09
Any lateral BML (%)	14
Any medial BML (%)	24
Lateral chondral defects score (possible range 0–8)	2.20 ± 0.91
Medial chondral defects score (possible range 0–8)	2.39 ± 1.09
Any lateral chondral defects (%)	44
Any medial chondral defects (%)	47
Any ROA of the knee at baseline (%)	16
Total ROA score at baseline (possible range 0–12)	0.3 ± 0.8
Knee alignment (degrees)	180.4 ± 2.6
Muscle strength (kg)	118.3 ± 48
WOMAC pain score (possible range 0–45)	3.7 ± 5.7

Where errors are shown, values are means ± SD. BML, bone marrow lesions; ROA, radiographic osteoarthritis; WOMAC, Western Ontario and McMaster University Osteoarthritis Index.

**Table 2:** Heritability estimates for the prevalence and severity of bone marrow lesions

Parameter	Step 1		Step 2		Step 3		Step 4	
	*h*^2^	*p*	*h*^2^	*p*	*h*^2^	*p*	*h*^2^	*p*
Lateral compartment								
Severity of BML	60 ± 26	0.01	53 ± 28	0.03	45 ± 28	0.06	45 ± 28	0.06
Prevalent BML	100	<0.01	100	0.02	99	0.04	99	0.05
Medial compartment								
Severity of BML	20 ± 25	0.21	65 ± 32	0.03	46 ± 31	0.07	56 ± 31	0.04
Prevalent BML	100	0.01	100	<0.01	99	0.02	99	0.01

Where errors are shown, values are means ± SD. BML, bone marrow lesions; *h*^2^, heritability estimate. In step 1, *h*^2 ^was estimated after adjustment for age, sex, height and weight; in step 2, further adjustment was made for muscle strength, knee pain and knee alignment; in step 3, further adjustment was made for chondral defects; in step 4, further adjustment was made for radiographic osteoarthritis.
